# The m^6^A-related gene signature for predicting the prognosis of breast cancer

**DOI:** 10.7717/peerj.11561

**Published:** 2021-06-04

**Authors:** Shanliang Zhong, Zhenzhong Lin, Huanwen Chen, Ling Mao, Jifeng Feng, Siying Zhou

**Affiliations:** 1 Center of Clinical Laboratory Science, The Affiliated Cancer Hospital of Nanjing Medical University & Jiangsu Cancer Hospital & Jiangsu Institute of Cancer Research, Nanjing, China; 2 Department of Pathology, The Affiliated Cancer Hospital of Nanjing Medical University & Jiangsu Cancer Hospital & Jiangsu Institute of Cancer Research, Nanjing, China; 3 Xinglin laboratory, The First Affiliated Hospital of Xiamen University, Nanjing, China; 4 Department of Thyroid Breast Surgery, The Affiliated Huai’an Hospital of Xuzhou Medical University, Huai’an, China; 5 Department of Medical Oncology, The Affiliated Cancer Hospital of Nanjing Medical University & Jiangsu Cancer Hospital & Jiangsu Institute of Cancer Research, Nanjing, China; 6 Department of General Surgery, The First Affiliated Hospital of Soochow University, Suzhou, China

**Keywords:** Breast cancer, Breast neoplasms, m6A modification, IGF2BP1, Prognosis

## Abstract

N6-methyladenosine (m^6^A) modification has been shown to participate in tumorigenesis and metastasis of human cancers. The present study aimed to investigate the roles of m^6^A RNA methylation regulators in breast cancer. We used LASSO regression to identify m^6^A-related gene signature predicting breast cancer survival with the datasets downloaded from Gene Expression Omnibus and The Cancer Genome Atlas (TCGA). RNA-Seq data of 3409 breast cancer patients from GSE96058 and 1097 from TCGA were used in present study. A 10 m^6^A-related gene signature associated with prognosis was identified from 22 m^6^A RNA methylation regulators. The signature divided patients into low- and high-risk group. High-risk patients had a worse prognosis than the low-risk group. Further analyses indicated that IGF2BP1 may be a key m^6^A RNA methylation regulator in breast cancer. Survival analysis showed that IGF2BP1 is an independent prognostic factor of breast cancer, and higher expression level of IGF2BP1 is associated with shorter overall survival of breast cancer patients. In conclusion, we identified a 10 m^6^A-related gene signature associated with overall survival of breast cancer. IGF2BP1 may be a key m^6^A RNA methylation regulator in breast cancer.

## Background

Breast cancer is the most common malignancy in women worldwide. Only in the United States, more than 250,000 new cases are diagnosed with breast cancer each year and 66,000 cases die from this malignancy (Siegel, Miller & Jemal, 2019). Besides environmental factors, the genetic background has been shown to associate with development of many cancers including breast cancer (Zhong et al., 2016). Although global gene expression patterns have been extensively studied in breast cancer, gene regulation at post-transcriptional level has not yet been fully investigated (Niu et al., 2019).

Several levels of post-transcriptional regulation can influence the gene expression in cells. For example, microRNAs (miRNAs) inhibit gene expression post-transcriptionally by either blocking translation or inducing degradation of messenger RNA (mRNA) targets (Zhong et al., 2013). Meanwhile, modifications such as the poly (A) tail and the 5′ cap have marked influence on gene expression. Over 150 different RNA modifications have been observed in various RNA molecules, including mRNAs, transfer RNAs (tRNAs), ribosomal RNAs (rRNAs), small non-coding RNAs and long non-coding RNAs (lncRNAs) (Yang et al., 2018). These RNA molecules can experience methylation at different positions, such as N6-methyladenosine (m^6^A), N1-methyladenosine, 5-methylcytosine, pseudouridine, N7-methyladenosine, and 2′-O-methylation (Chai et al., 2019). Of these modifications, m^6^A was first discovered in the 1970s and is the most prevalent modification in the mRNA (Desrosiers, Friderici & Rottman, 1974). Three classes of m^6^A regulatory enzymes are referred to as ‘writers’, ‘erasers’ and ‘readers’ (Heck & Wilusz, 2019). ‘writers’, such as RBM15/15B (Meyer & Jaffrey, 2017), METTL3 (Schumann, Shafik & Preiss, 2016), METTL14 (Liu et al., 2014), WTAP (Ping et al., 2014) and KIAA1429 (Schwartz et al., 2014) catalyze the formation of m^6^A; ‘erasers’, ALKBH5 (Zheng et al., 2013) and FTO (Jia et al., 2011), remove m^6^A from select transcripts; and ‘readers’ such as HNRNPA2B1 (Alarcon et al., 2015), YT521-B homology (YTH) domain-containing proteins (YTHDF1, YTHDF2, YTHDF3, YTHDC1 and YTHDC2) (Liao, Sun & Xu, 2018), HNRNPC (Liu et al., 2015) and HNRNPG (Liu et al., 2017) recognize and generate functional signals.

M^6^A RNA modifications participate in the tumorigenesis and metastasis of multiple malignancies by regulating RNA transcript, splicing, processing and translation (Chen, Zhang & Zhu, 2019). For example, Wu et al. (2019) found that reduced m^6^A may contribute to tumorigenesis and is associated with poor prognosis in breast cancer. In the present study, we comprehensively analyzed the expression of previous reported m^6^A RNA regulators in breast cancer using the data from Gene Expression Omnibus (GEO) and The Cancer Genome Atlas (TCGA).

## Material and Methods

### Selection of m^6^A RNA methylation regulators

After searching published literature, we obtained a list of 22 m^6^A RNA methylation regulators: RBM15/15B (Meyer & Jaffrey, 2017), METTL3 (Schumann, Shafik & Preiss, 2016), METTL14 (Liu et al., 2014), METTL16 (Warda et al., 2017), WTAP (Ping et al., 2014), KIAA1429 (also known as VIRMA) (Schwartz et al., 2014), ALKBH5 (Zheng et al., 2013), FTO (Jia et al., 2011), IGF2BP1/2/3 (Huang et al., 2018b), HNRNPA2B1 (Alarcon et al., 2015), YTH domain-containing proteins (YTHDC1 YTHDC2, YTHDF1, YTHDF2 and YTHDF3) (Liao, Sun & Xu, 2018), HNRNPC (Liu et al., 2015), HNRNPG (also known as RBMX) (Liu et al., 2017), RBMX (Liu et al., 2017), FMR1 (Edupuganti et al., 2017), EIF3 (Meyer et al., 2015).

### Data acquisition

We downloaded clinical data for 1097 female breast cancer patients as well as their level 3 RNA-Seq data (HTSeq-Counts) from TCGA (accessed December 2019) (search term: BRCA) (Cancer Genome Atlas, 2012; Tomczak, Czerwinska & Wiznerowicz, 2015). Dataset of GSE96058 for 3409 breast cancer patients was downloaded from GEO (accession number: GSE96058) (Brueffer et al., 2018).

### Data analysis

All of the analyses in present study were done with R software (version 4.0.1). Because GSE96058 includes 3409 patients and detailed clinical information, we investigated the relationships between regulators and pathological features of breast cancer, including molecular classification [Normal, Luminal A (LumA), Luminal B (LumB), Her2 and Basal] and Nottingham histologic grade (grade2, grade3 and grade4) with GSE96058. R package of pheatmap (R package version 1.0.12) was used to plot heatmap. Box plot was drawn using ggpubr package (R package version 0.2.4). R package of “beeswarm” (R package version 0.2.3) was used to show the expression level of each gene in different groups.

To analyze the RNA-Seq data from TCGA, raw read counts were normalized and the differentially expressed genes were compared between breast cancer tissues and adjacent normal tissues using DESeq2 (Love, Huber & Anders, 2014) (R package version 1.18.1). The data of breast cancer tissues or adjacent normal tissues were identified according to their TCGA barcode (https://docs.gdc.cancer.gov/Encyclopedia/pages/TCGA_Barcode/). Adjacent normal tissues were taken from greater than two cm from the tumor (Cancer Genome Atlas, 2012). We used the Benjamini & Hochberg method (Benjamini et al., 2001) to correct for multiple testing. Fold-change >2 and adjusted *P* value < 0.05 were used as selection criteria to identify differentially expressed genes between different groups. Then, the differentially expressed m^6^A RNA methylation regulators were selected from the differentially expressed genes.

Most useful prognostic markers were selected from m^6^A RNA methylation regulators using LASSO (Tibshirani, 1996) penalized regression analysis. LASSO is a feature selection method to nullify the impact of irrelevant features. With increasing *λ*, LASSO shrinks all regression coefficients towards zero and sets the coefficients of irrelevant features exactly to zero. The optimal *λ* is selected as the *λ* that yields minimum cross validation error in 10-fold cross validation. GSE96058 was used as the training set, and TCGA was used as the test set. The risk score was calculated for each subject by sum of the product of expression level of each gene and the corresponding regression coefficients as in our previous study (Liu et al., 2019). We used the “glmnet” package (R package version 2.0-16) to conduct the LASSO analysis and a *P* value < 0.05 was considered statistically significant.

In survival analyses, patients were separated into two groups according to the expression level of a gene or the risk scores, and the median was used as cut-off. Then, the log-rank test was used to assess the overall survival (OS) with the survival package (R package version 3.1-7). Hazard ratios (HRs) and their 95% confidence intervals (CIs) were calculated using Cox proportional hazards. We used forestplot package (R package version 3.4.3) to draw forest plot to show the HRs and 95% CIs of the genes.

To identify downstream genes of m^6^A RNA methylation regulators, the correlations between m^6^A RNA methylation regulators and other genes were assessed with the RNA-Seq data from GSE96058 and TCGA using Spearman’s correlation. The Spearman’s rho was used to assess the degree of association between IGF2BP1 and its potential coexpressed genes. Only the coexpressed genes identified from the both datasets were kept.

We used clusterProfiler package (Yu et al., 2012) (R package version 4.0.1) to carry out Kyoto Encyclopedia of Genes and Genomes (KEGG) and Gene Ontology (GO) enrichment analyses. Benjamini & Hochberg method was applied to correct for multiple testing (Benjamini et al., 2001). An adjusted *P* value of less than 0.05 was considered statistically significant. A network of genes and enriched GO terms was constructed with Cytoscape software (version 3.8) (Shannon et al., 2003). GOplot package was utilized to illustrate the relationship between enriched KEGG pathways and genes.

## Results

### Expression of m^6^A RNA methylation regulators in breast cancer

**Figure 1:**
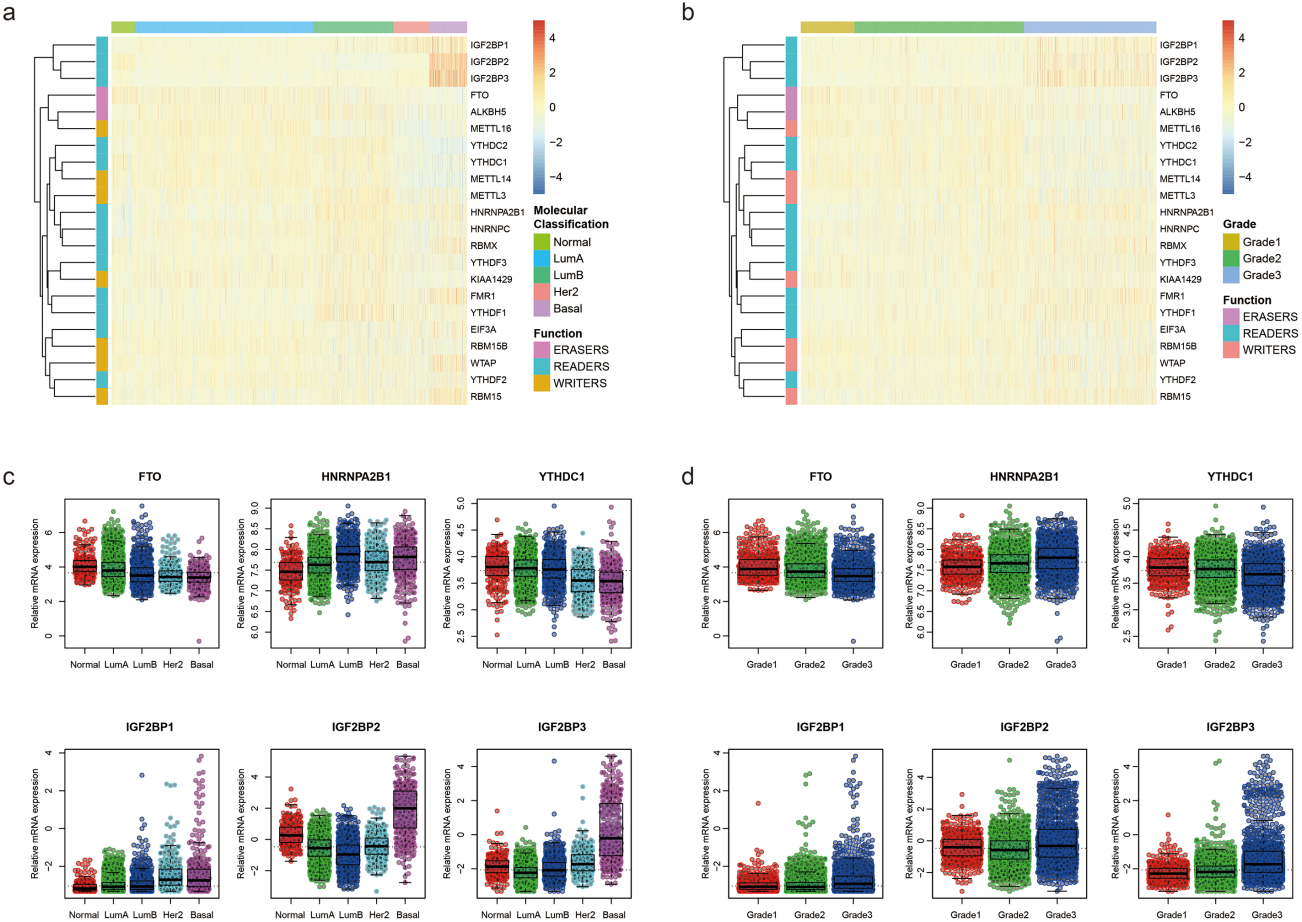
Expression of the 22 m^6^A RNA methylation regulators in GSE96058. (A) Heatmap of the relative expression level of the 22 m^6^A RNA methylation regulators across all 3409 patients in GSE96058. Patients are categorized as normal or by the molecular classification of their breast cancer Luminal A (Lum A), Luminal B (lum B), Her2 or basal. The 22 m^6^A methylation regulators are clustered into groups with similar expression behavior in the five different categories. The functional group of the m^6^A methylation regulator is also indicated. (B) Heatmap of the relative expression level of the 22 m^6^A RNA methylation regulators across all 3409 patients in GSE96058. Patients are categorized by grade. The 22 m^6^A methylation regulators are clustered into groups with similar expression behavior in the three categories. (C) The relative expression level of 6 representative m6A RNA methylation regulators in different molecular subtypes of breast cancer. The expression level of the six regulators shows a decreased or increased trend with the increase of malignancy of breast cancer from normal to basal. (D) The relative expression levels of six representative m^6^A RNA methylation regulators in different grades of breast cancer. The expression level of the six regulators shows a decreased or increased trend with grade from 1 to 3.

To uncover the biological functions of these m^6^A RNA methylation regulators, we explored the relationships between each regulator and pathological features of breast cancer, including molecular classification (Normal, LumA, LumB, Her2 and Basal) and Nottingham histologic grade (grade1, grade2 and grade3) with GSE96058. The expression level of each m^6^A RNA methylation regulator according to molecular classification is presented in Fig. 1A, showing that most m6A RNA methylation regulators had different expression levels in different molecular classifications. Similar results were found in different grades (Fig. 1B). We used beeswarm plots to show distributions of the regulators in different molecular classifications and grades (Figs. 1C, S1, 1D and S2). With the increase of malignancy of breast cancer, the expression level of several m^6^A RNA methylation regulators, such as FTO, HNRNPA2B1 YTHDC1, IGF2BP1, IGF2BP2 and IGF2BP3, decreased or increased (Fig. 1C). The expression level of these six m^6^A RNA methylation regulators also had a trend with grade from 1 to 3 (Fig. 1D).

**Figure 2:**
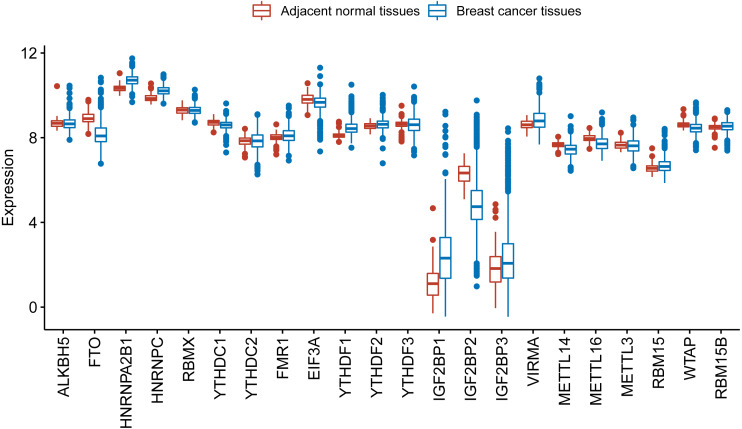
Expression level of the 22 m^6^A RNA methylation regulators in breast cancer tissues and adjacent normal tissues in TCGA. The box plot shows the distribution of normalized read count based on the five number summary: minimum, first quartile, median, third quartile, and maximum. The central rectangle spans the first quartile to the third quartile. The segment inside the rectangle shows the median and “whiskers” above and below the box show the locations of the minimum and maximum. The dots are outliers.

We further used TCGA RNA-Seq data to explore whether there is a difference in expression levels of m^6^A RNA methylation genes between adjacent normal tissues and breast cancer tissues. We identified 6168 differentially expressed genes after comparing 1097 breast cancer tissues with 113 adjacent normal tissues. Then, we identified 17 m^6^A RNA methylation regulators had an adjusted *P* value less than 0.05, including 2 (IGF2BP1 and IGF2BP3) with a fold change > 2 (Table S1). Figure 2 shows the distribution of expression levels of the 22 m^6^A RNA methylation genes in breast cancer tissues and adjacent normal tissues.

### Identification of an m^6^A-related gene signature associated with prognosis

**Figure 3:**
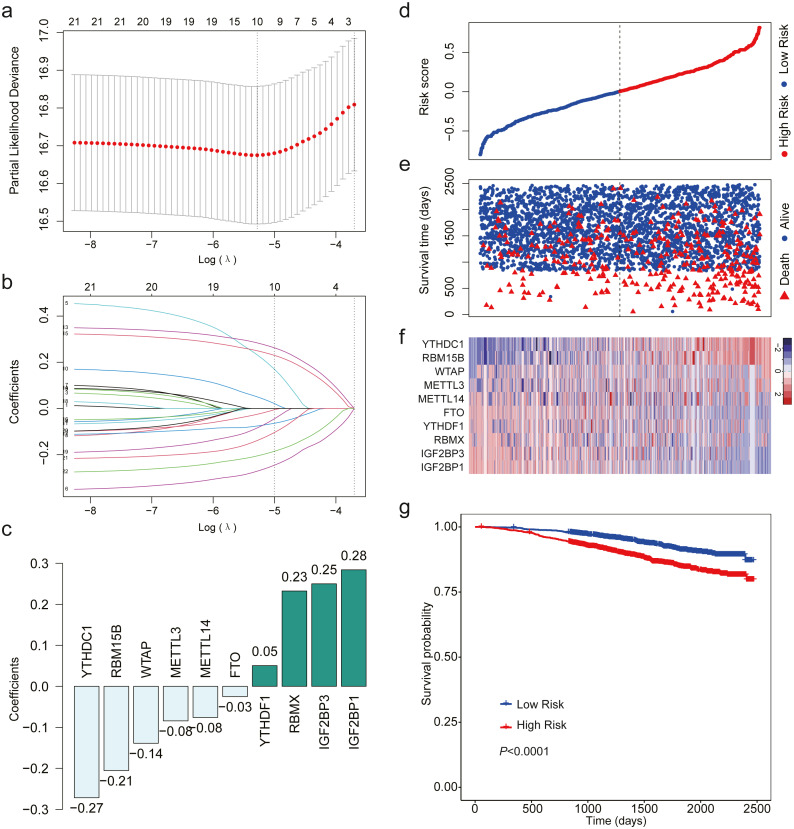
LASSO Cox regression of the 22 m^6^A RNA methylation regulators using GSE96058. (A) Ten m^6^A RNA methylation regulators were selected by LASSO Cox regression analysis. The top *x*-axis shows the number of regulators with non-zero coefficients. The optimal *λ* is selected as the *λ* that yields minimum cross validation error in 10-fold cross validation. (B) LASSO coefficient profiles of the 22 m^6^A RNA methylation regulators. The top *x*-axis shows the number of regulators with a non-zero coefficient. With increasing *λ*, LASSO shrinks all regression coefficients towards zero and sets the coefficients of irrelevant features exactly to zero. Ten regulators with non-zero coefficients is selected at optimal *λ*. (C) Ten m^6^A RNA methylation regulators and their coefficients. (D) The distribution of the risk score for the patients. (E) Survival time and status of the patients in the high- and low-risk groups, as defined by the risk score. (F) Expression patterns of the 10 m^6^A RNA methylation regulators. (g) Patients with high risk score had shorter overall survival time than those with low risk score (HR = 1.907, 95% CI [1.533–2.373]). Dotted line in (D) and (E) represents the median of the risk score. The patients in (D), (E) and (F) were sorted by risk score in ascending order.

After analyzing the expression level of the 22 m^6^A RNA methylation regulators using LASSO regression analysis, a 10 m^6^A-related gene signature associated with OS was identified in breast cancer patients (Figs. 3A–3C). We calculated risk scores for all the patients. When we sorted the patients according to the risk score, it was easily noted that high-risk group had more deaths than low risk group (Figs. 3D–3E). The heatmap indicated that the expression level of the 10 m^6^A-related genes showed an increase or decrease trend from low risk score to high risk score (Fig. 3F). Then, we analyzed the over survival of breast cancer patients according to the risk score, and found that OS of high-risk patients was shorter OS than that of low risk patients (HR = 1.907, 95% CI [1.533–2.373]; Fig. 3G).

### Validation of the m^6^A-related gene signature using data from TCGA

**Figure 4:**
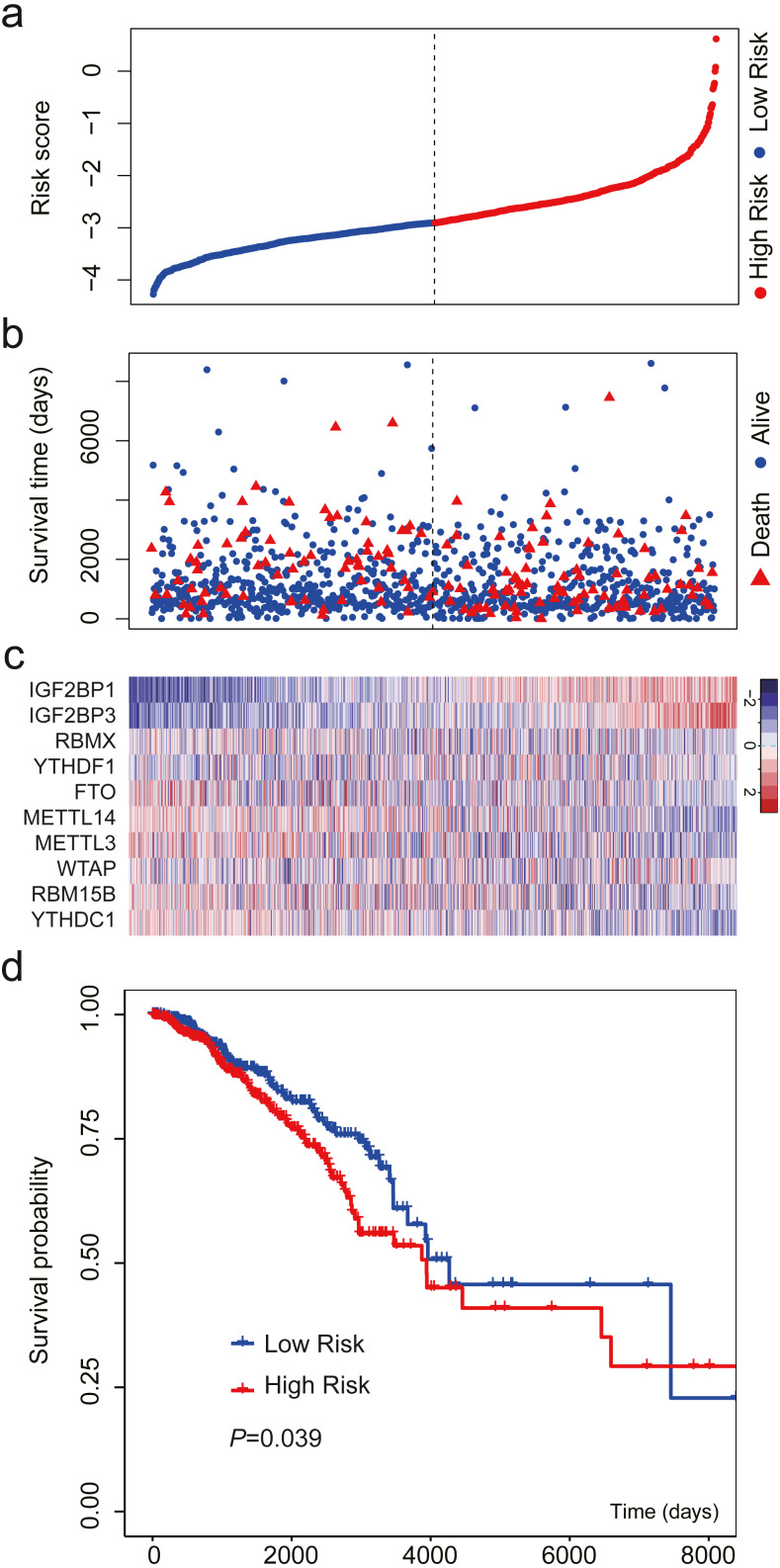
Validation of the m^6^A-related gene signature using data from TCGA. (A) The distribution of the risk score for the patients. (B) Survival time and status of the patients in the high- and low-risk groups, as defined by the risk score. (C) Expression patterns of the 10 m^6^A RNA methylation regulators. (D) Patients with high risk score had shorter overall survival time than those with low risk score (HR = 1.406, 95% CI [1.016–1.946]). The dotted line in (A) and (B) represents the median of the risk score. The patients in (A), (B) and (C) were sorted by risk score in ascending order.

We validated the m^6^A-related gene signature using the data from TCGA. The results were similar (Fig. 4). When the patients were sorted by risk score and divided into two groups, we found that high-risk patients had more deaths and shorter OS than low-risk patients (Figs. 4A–4B). The expression level of the 10 m^6^A-related genes also showed a trend from low risk score to high risk score (Fig. 4C). The results of survival analysis indicated that high-risk patients had a HR of 1.406 (95%: 1.016−1.946; Fig. 4D).

### M^6^A RNA methylation regulators associated with breast cancer survival

**Figure 5:**
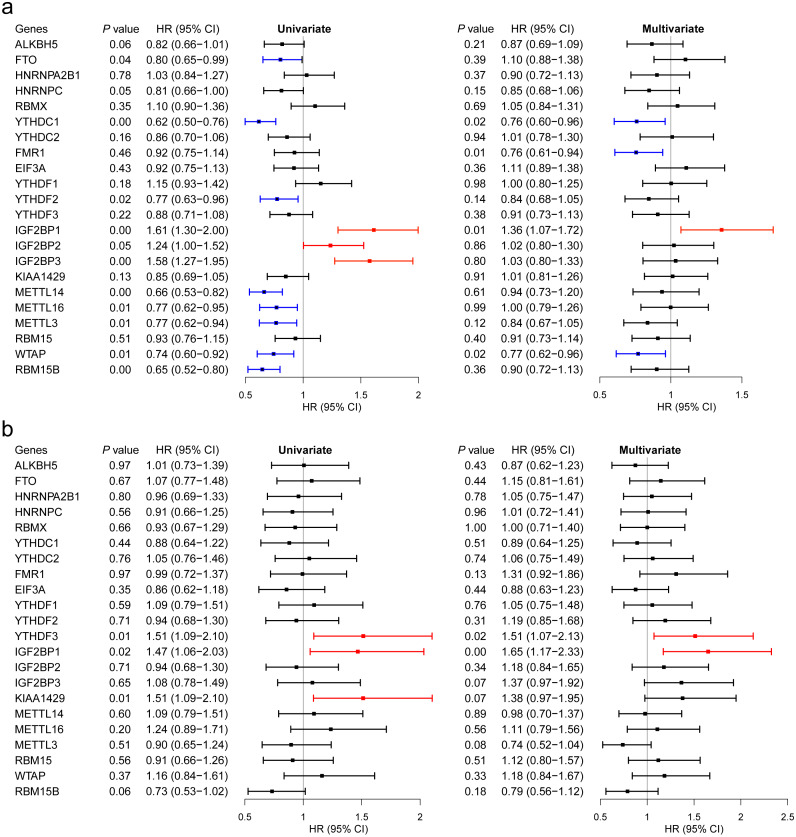
Survival analysis of the 22 m^6^A RNA methylation regulators in breast cancer patients. (A) Survival analysis was conducted using GSE96058. (B) Survival analysis was conducted using TCGA.

We then evaluated the association between the expression levels of the m^6^A RNA methylation genes and breast cancer survival. Regarding GSE96058, 11 regulators had significant associations in univariate analysis, including 8 beneficial regulators and harmful regulators (Fig. 5A). However, after adjusted to age, molecular classification, grade, tumor size, lymph node status, endocrine treated, and chemotherapy treated, only YTHDC1, FMR1, IGF2BP1 and WTAP showed a significant association (Fig. 5A).

With respect to TCGA, higher expression levels of YTHDF3, IGF2BP1 and KIAA1429 were associated with shorter OS of breast cancer patients in univariate analysis (Fig. 5B). After adjusted for age and stage, YTHDF3 and IGF2BP1 still had a significant association with OS (Fig. 5B). Combining the findings in Fig. 2, IGF2BP1 may be a key m^6^A RNA methylation regulator associated with OS of breast cancer patients. Therefore, we have further explored the potential mechanisms by which IGF2BP1 exerts its effect.

### Genes coexpressed with m^6^A RNA methylation regulators

Totally, 182 coexpressed genes with a Spearman’s rho larger than 0.3 were identified for IGF2BP1 from GSE96058 and TCGA (Table S2).

**Figure 6:**
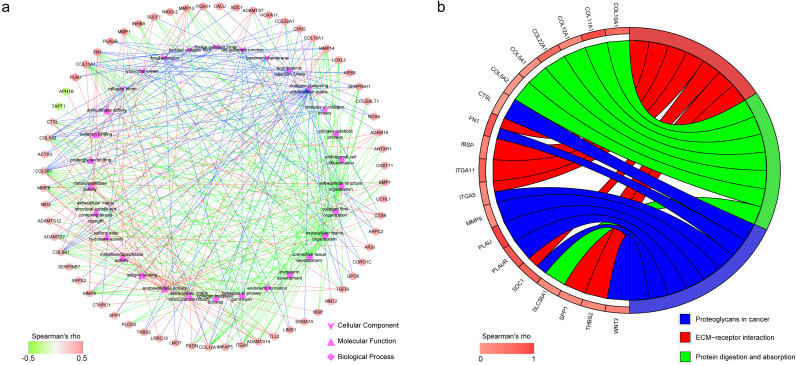
Functional annotation of the 182 coexpressed genes of IGF2BP1. (A) The top 10 gene ontology terms in biological process, molecular function and cellular component as well as the related coexpressed genes. (B) KEGG enrichment analysis of the coexpressed genes.

In GO enrichment analysis, genes were annotated to 3 ontologies: biological process (BP), molecular function (MF) and cellular component (CC). Figure 6A presents the coexpressed genes and the top 10 GO terms in BP, MF and CC. Collagen-containing extracellular matrix, endoplasmic reticulum lumen, and focal adhesion were the 3 most commonly assigned terms for the CC. Endopeptidase activity, extracellular matrix structural constituent, and metalloendopeptidase activity were the most commonly assigned terms for the MF. The most commonly assigned GO terms in the BP were extracellular matrix organization, extracellular structure organization, and connective tissue development.

The result of KEGG enrichment analysis is presented in Fig. 6B. The 19 coexpressed genes are enriched 3 pathways including proteoglycans in cancer, ECM–receptor interaction, and protein digestion and absorption.

## Discussion

In this study, we identified a 10 m^6^A-related gene signature associated with breast cancer OS using RNA-Seq data from GSE96058 and TCGA. The 10 m^6^A-related gene signature divided patients into low- and high-risk group, and survival time of high-risk patients was shorter than that of low-risk group. Then, we evaluated the expression level of previous reported m^6^A RNA genes in breast cancer. Our results showed that m^6^A RNA methylation regulators have different expression characteristics according to molecular classification and grade of breast cancer. IGF2BP1 may be a key m^6^A RNA methylation regulator associated with OS of breast cancer patients.

We found m^6^A RNA methylation regulators showed different expression characteristics in different molecular classifications and grades of breast cancer. The expression level of HNRNPA2B1, IGF2BP1, IGF2BP2 and IGF2BP3 was shown to increase as malignancy increased, but FTO and YTHDC1 were reduced as malignancy increased. After comparing breast cancer tissues with adjacent normal tissues, we found IGF2BP1 and IGF2BP3 had a fold change >2 in breast cancer tissues. Although IGF2BP1-3 were expressed in a lower level than other m^6^A RNA methylation regulators, they had a higher level in more malignant cancer. In the survival analyses, we found that IGF2BP1 is an independent prognostic factor of breast cancer. IGF2BP1 may be a key m^6^A RNA methylation regulator associated with OS of breast cancer patients.

The full name of IGF2BP1 is insulin-like growth factor-2 mRNA-binding protein 1, which expresses in more than 16 cancers and most fetal tissues but only in a limited number of normal adult tissues (Huang et al., 2018c). IGF2BP1 can act as m6A readers to regulate more than 3000 transcript targets (Dominissini et al., 2012). After the target mRNAs are methylated at the 3′-UTR, IGF2BP1 can recognize it and inhibit the decay of m^6^A-RNAs under the co-effects of stabilizers such as ELAVL1 (Huang et al., 2018a). IGF2BP1 can also compete for the same m6A sites with other m6A readers such as YT521-B homology (YTH) domain containing proteins (YTHDFs). YTHDFs act as m6A readers and promote degradation of modified RNA (Huang et al., 2018c). Therefore, we used Spearman’s rank analysis to identify potential coexpression genes which may be regulated by IGF2BP1. The result showed that most genes had a positive correlation with IGF2BP1. IGF2BP1 has been shown to implicate in proliferation, migration, invasion, adhesion, and apoptosis of tumor cells (Bell et al., 2013). In the GO annotation, we found the coexpressed genes of IGF2BP1 were mainly enriched in the terms associated with extracellular matrix, adhesion, and collagen metabolism. KEGG enrichment analysis indicated that these coexpressed genes were enriched in ECM-receptor interaction, protein digestion and absorption, and proteoglycans in cancer. ECM-receptor interaction has been shown to play important roles in breast cancer (Bao et al., 2019). Protein digestion and absorption, and proteoglycans in cancer are two pathways related to extracellular matrix and microenvironment of tumor cells (Afratis et al., 2017). Based on the above results, we proposed that IGF2BP1 up-regulates the target genes’ expression, thus the destroying extracellular matrix, inhibiting apoptosis, and promoting migration, adhesion and proliferation, and finally promotes the progression of breast cancer. Therefore, IGF2BP1 may serve as a target for breast cancer treatment.

In conclusion, a 10 m^6^A-related gene signature associated with OS was identified in breast cancer patients. The signature divided patients into low- and high-risk group. High-risk patients had shorter survival time than low-risk group. The 10 m^6^A-related gene signature may be used in clinical practice to predict the survival of breast cancer patients in the future. We then systematically analyzed the expression of 22 m^6^A RNA regulators in breast cancer and identified that IGF2BP1 may be a key m^6^A RNA methylation regulator associated with OS of breast cancer patients. Further studies are needed to validate the 10 m^6^A-related gene signature in larger samples and confirm the roles of IGF2BP1 in breast cancer using experimental methods.

##  Supplemental Information

10.7717/peerj.11561/supp-1 Supplemental Information 1 The expression levels of 16 m^6^A RNA methylation regulators in different molecular subtypes of breast cancer

10.7717/peerj.11561/supp-2 Supplemental Information 2 The expression levels of 16 m^6^A RNA methylation regulators in different grades of breast cancer

10.7717/peerj.11561/supp-3 Supplemental Information 3 Expression of the 22 m6A RNA methylation regulators in breast cancer tissues compared with that in adjacent normal tissues in TCGA

10.7717/peerj.11561/supp-4 Supplemental Information 4 Spearman’s correlation of IGF2BP1 and the genes with a correlation of at least 0.3

## Additional Information and Declarations

### Data Availability

The following information was supplied regarding data availability: All the raw data is available at TCGA (search term: BRCA) and GEO: GSE96058.
