# Supplementary figures and images for: The m6A-related gene signature for predicting the prognosis of breast cancer
Source: PeerJ. 2021 Jun 4;9:e11561. doi: 10.7717/peerj.11561 (PMC8183431; doi:10.7717/peerj.11561)

**ALKBH5**

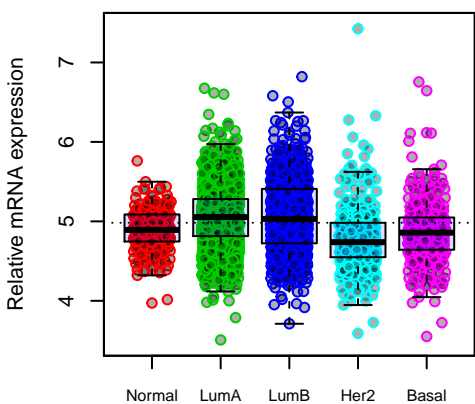

**HNRNPC**

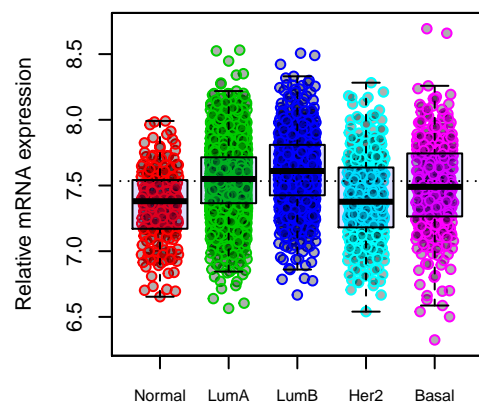

**RBMX**

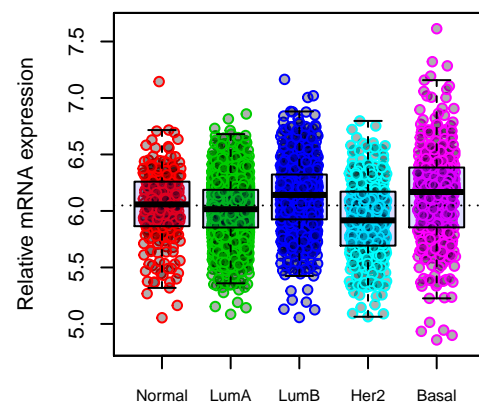

**YTHDC2**

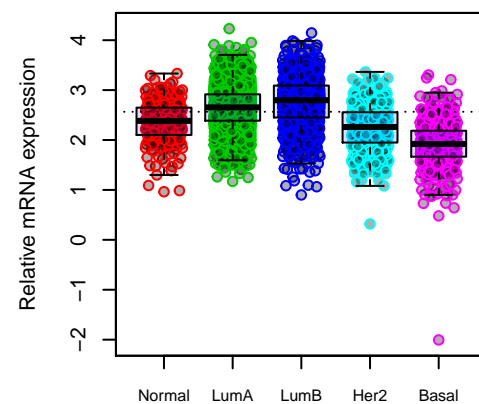

**FMR1**

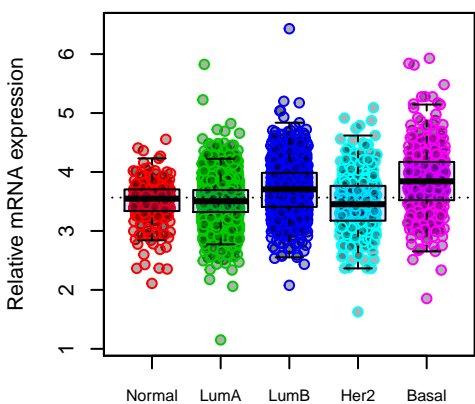

**EIF3A**

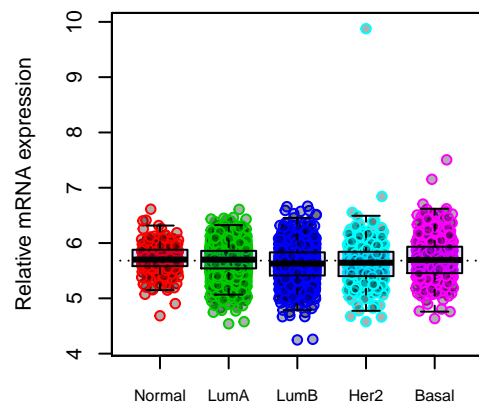

**YTHDF1**

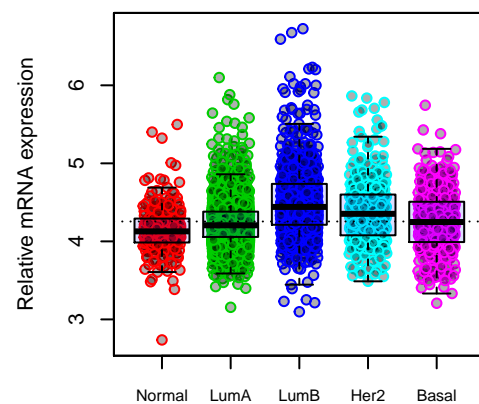

**YTHDF2**

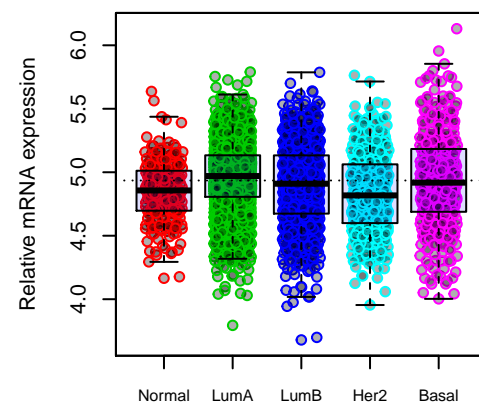

**YTHDF3**

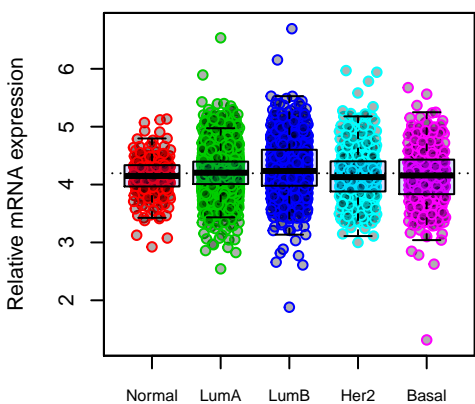

**KIAA1429**

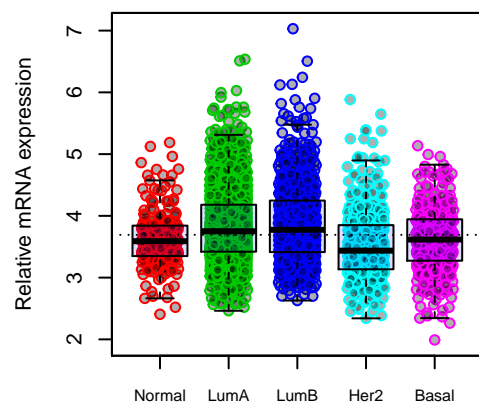

**METTL14**

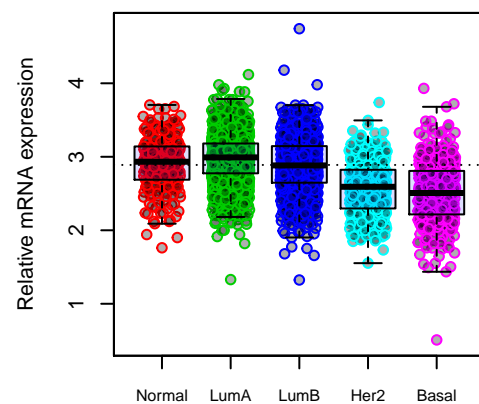

**METTL16**

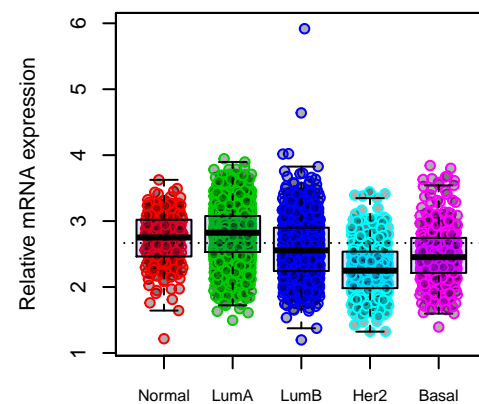

**METTL3**

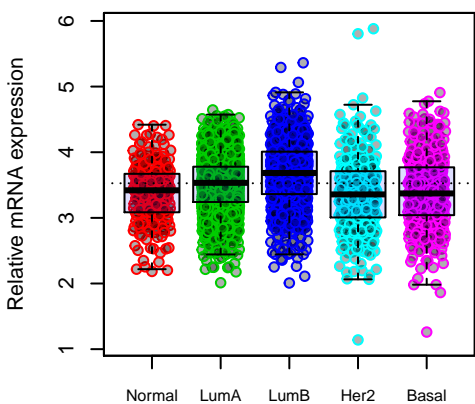

**RBM15**

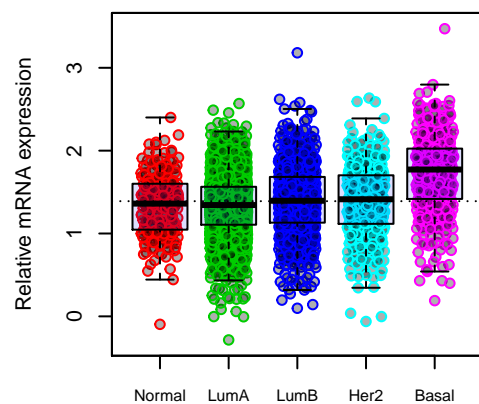

**WTAP**

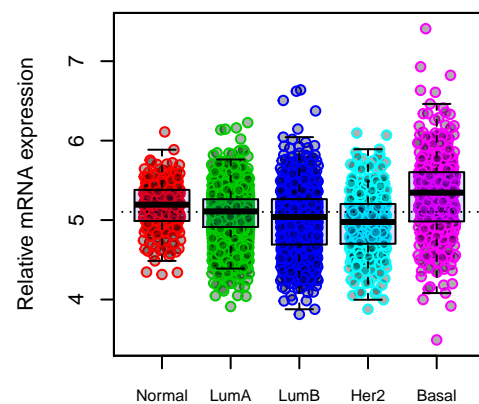

**RBM15B**

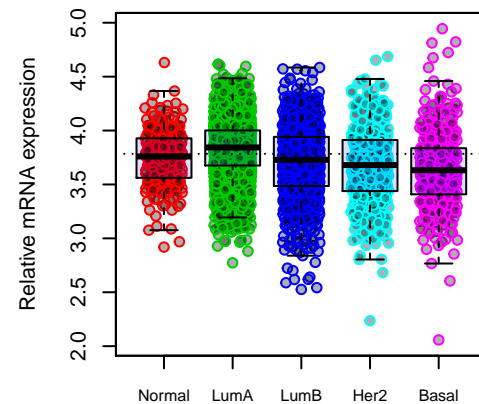

Supplement: Supplemental Information 1 [file peerj-09-11561-s001.pdf]

**ALKBH5**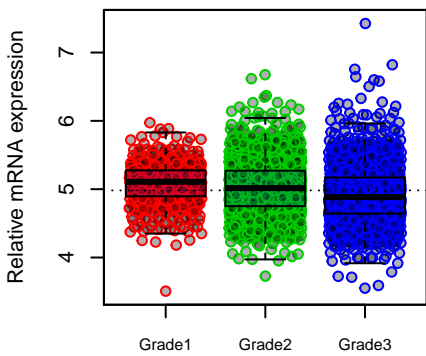**HNRNPC**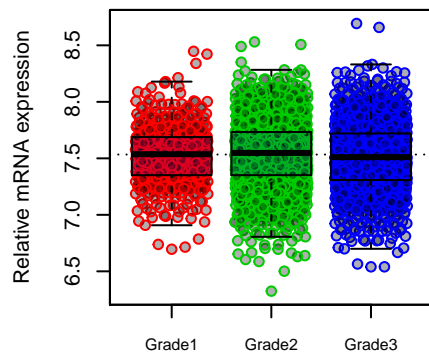**RBMX**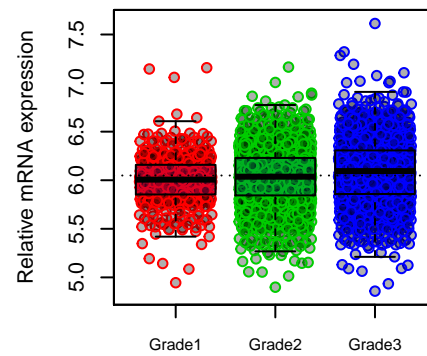**YTHDC2**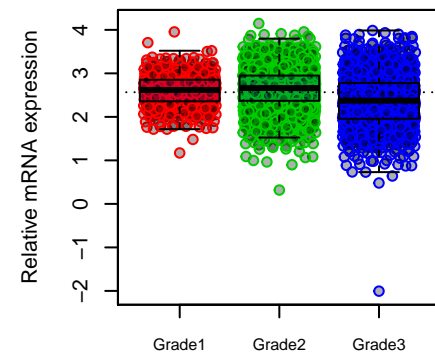**FMR1**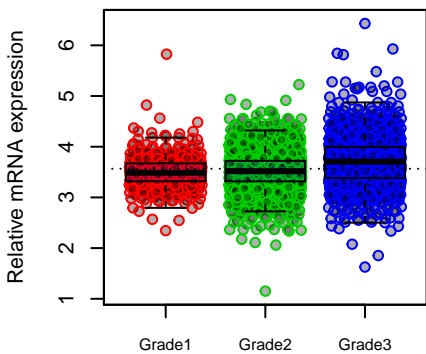**EIF3A**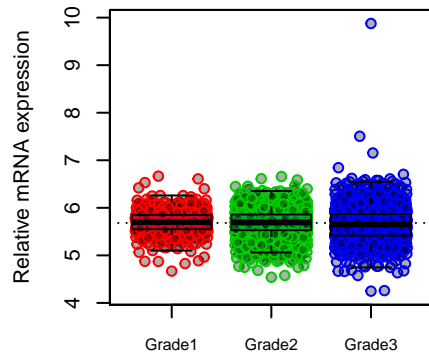**YTHDF1**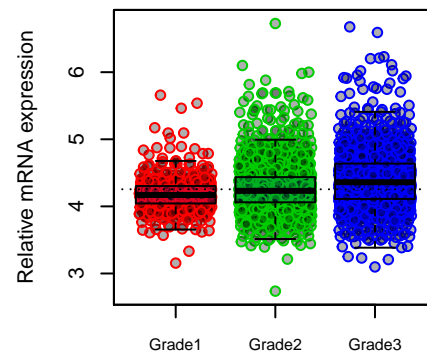**YTHDF2**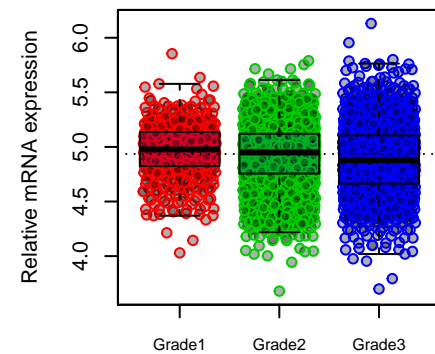**YTHDF3**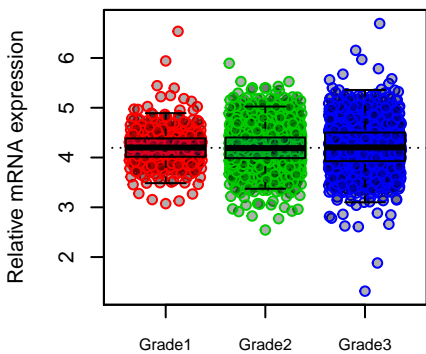**KIAA1429**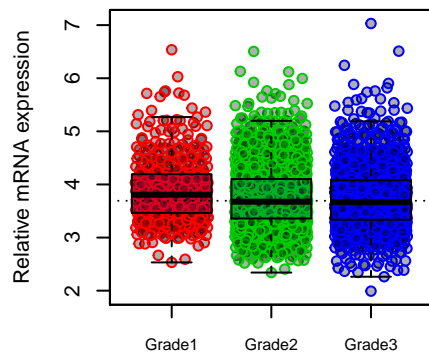**METTL14**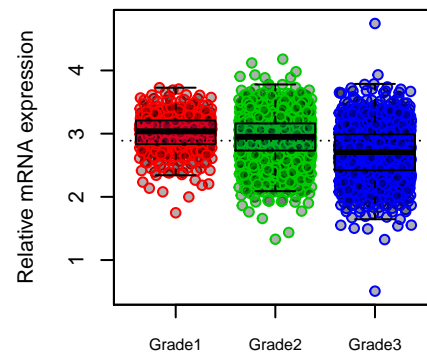**METTL16**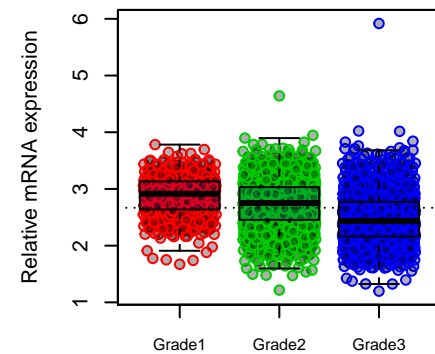**METTL3**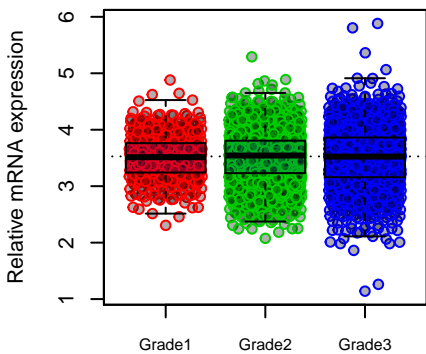**RBM15**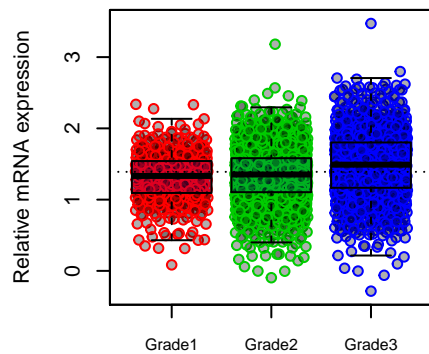**WTAP**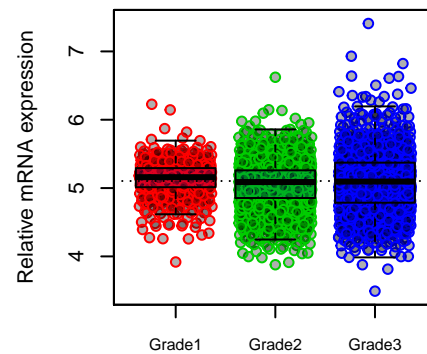**RBM15B**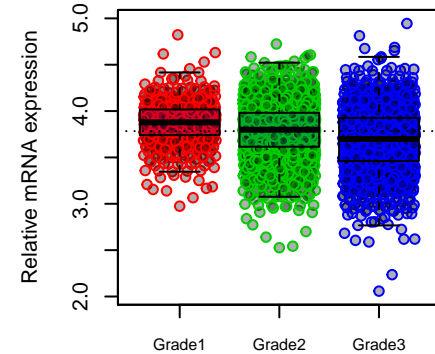

Supplement: Supplemental Information 2 [file peerj-09-11561-s002.pdf]
