# Supplementary material for: The m6A-related gene signature for predicting the prognosis of breast cancer
Source: PeerJ. 2021 Jun 4;9:e11561. doi: 10.7717/peerj.11561 (PMC8183431; doi:10.7717/peerj.11561)
Supplement: Supplemental Information 3 [file peerj-09-11561-s003.pdf]

Table S1 Expression of the 22 m6A RNA methylation regulators in breast cancer tissues compared with that in adjacent normal tissues in TCGA.

Genes	Log2 (Fold Change)	*P* value	P_{BH} value*
ALKBH5	0.00	9.77E-01	1.00E+00
FTO	-0.91	1.13E-29	6.75E-29
HNRNPA2B	0.58	3.43E-66	6.54E-65
HNRNPC	0.49	4.22E-56	6.11E-55
RBMX	0.02	5.72E-01	6.60E-01
YTHDC1	-0.16	7.50E-08	1.66E-07
YTHDC2	0.08	1.54E-01	2.00E-01
FMR1	0.23	5.60E-06	1.10E-05
EIF3A	-0.23	7.29E-06	1.41E-05
YTHDF1	0.61	8.71E-40	7.50E-39
YTHDF2	0.15	3.97E-05	7.32E-05
YTHDF3	0.10	8.71E-02	1.17E-01
IGF2BP1	3.01	3.83E-47	4.19E-46
IGF2BP2	-0.42	2.33E-02	3.36E-02
IGF2BP3	2.27	2.00E-21	8.53E-21
VIRMA	0.49	1.63E-12	4.68E-12
METTL14	-0.27	8.81E-10	2.18E-09
METTL16	-0.31	6.24E-12	1.74E-11
METTL3	-0.01	8.42E-01	9.14E-01
RBM15	0.21	1.11E-05	2.13E-05
WTAP	-0.19	6.66E-06	1.30E-05
RBM15B	0.10	5.51E-03	8.49E-03

*, Adjusted for multiple testing using Benjamini & Hochberg method.
