# Supplementary material for: The m6A-related gene signature for predicting the prognosis of breast cancer
Source: PeerJ. 2021 Jun 4;9:e11561. doi: 10.7717/peerj.11561 (PMC8183431; doi:10.7717/peerj.11561)
Supplement: Supplemental Information 4 [file peerj-09-11561-s004.pdf]

Table S2 Spearman's correlation of IGF2BP1 and the genes with a correlation of at least 0.3

	TCGA		GSE96058	
Genes	*P* value	rho	*P* value	rho
PLAU	1.08E-62	0.48	4.34E-230	0.51
CEMIP	1.63E-56	0.45	2.53E-213	0.50
ERMN	2.78E-44	0.41	6.40E-212	0.50
COL11A1	2.32E-51	0.43	8.57E-209	0.49
ULBP2	7.34E-68	0.49	3.61E-192	0.48
FN1	1.15E-53	0.44	7.82E-187	0.47
PLAUR	3.39E-44	0.40	1.25E-186	0.47
ALPK2	1.17E-49	0.43	4.14E-186	0.47
MMP1	1.28E-64	0.48	1.42E-184	0.47
C5orf46	1.83E-39	0.38	1.12E-181	0.46
INHBA	1.90E-39	0.38	8.97E-180	0.46
SULF1	1.45E-41	0.39	7.44E-179	0.46
NKX3-2	4.55E-40	0.39	1.12E-177	0.46
MMP13	2.61E-48	0.42	1.60E-177	0.46
SUGCT	5.00E-38	0.38	1.91E-177	0.46
DCBLD1	1.44E-40	0.39	4.07E-174	0.46
ITGA11	1.99E-36	0.37	7.75E-167	0.45
CALU	4.32E-46	0.41	1.12E-165	0.45
SDC1	1.34E-35	0.36	2.56E-163	0.44
HSD17B6	7.19E-29	0.33	2.33E-160	0.44
ADAMTS7	6.74E-31	0.34	5.87E-160	0.44
TMEM158	3.85E-35	0.36	1.83E-156	0.43
HOXA11	1.14E-35	0.37	6.51E-155	0.43
APBA2	4.06E-44	0.40	1.55E-153	0.43
EPYC	1.74E-49	0.43	3.59E-153	0.43
NTNG2	9.98E-28	0.32	8.35E-151	0.43
COL22A1	4.31E-39	0.38	2.53E-150	0.43
BCAT1	2.74E-60	0.47	5.31E-150	0.43
CDH2	4.48E-45	0.41	8.29E-150	0.43
COL10A1	6.27E-30	0.33	1.84E-148	0.42
ULBP1	5.20E-59	0.46	5.18E-143	0.42
NRP2	6.59E-45	0.41	6.02E-143	0.42
HHIPL1	3.63E-35	0.36	9.89E-142	0.41
SOX11	3.70E-35	0.36	1.50E-141	0.41
CSMD2	6.79E-45	0.41	1.33E-139	0.41
TM6SF2	8.45E-28	0.32	3.35E-138	0.41
SGIP1	1.20E-36	0.37	8.16E-138	0.41
CLDN14	5.35E-47	0.42	2.14E-137	0.41
MMP14	7.26E-32	0.35	4.31E-137	0.41
FNDC1	4.93E-47	0.42	6.40E-137	0.41
SLC16A3	4.85E-27	0.32	1.65E-133	0.40
LOXL2	5.91E-38	0.38	2.93E-133	0.40
ARSB	1.63E-48	0.42	6.47E-133	0.40
SERPINH1	2.18E-35	0.36	4.81E-132	0.40

PPEF1	3.74E-26	0.31	1.11E-131	0.40
TREM1	1.48E-48	0.42	1.22E-131	0.40
COLGALT1	8.06E-33	0.35	1.45E-130	0.40
NOX4	5.96E-32	0.35	6.96E-129	0.40
TRIM59	7.47E-35	0.36	1.97E-128	0.40
SALL4	5.75E-44	0.40	1.73E-127	0.39
SLC38A5	1.35E-32	0.35	2.01E-126	0.39
MTHFD1L	1.69E-37	0.37	4.53E-126	0.39
ADAM19	6.00E-55	0.45	1.01E-125	0.39
NETO1	1.57E-47	0.42	2.82E-125	0.39
EN1	1.11E-36	0.37	9.27E-125	0.39
LOC100507600	3.27E-46	0.41	1.07E-124	0.39
ANTXR1	1.41E-27	0.32	2.44E-124	0.39
CLTCL1	1.81E-34	0.36	8.53E-124	0.39
CHST11	1.46E-45	0.41	1.27E-123	0.39
GJB2	7.63E-36	0.37	2.44E-123	0.39
BMP1	6.43E-28	0.32	1.08E-122	0.39
UCHL1	4.41E-33	0.35	1.28E-122	0.39
CTSB	7.94E-33	0.35	7.37E-121	0.38
CHST6	1.00E-41	0.39	1.17E-120	0.38
ALDH1B1	2.28E-29	0.33	4.48E-120	0.38
NINJ2	1.71E-28	0.33	7.46E-118	0.38
ARPC2	3.27E-27	0.32	1.93E-116	0.38
ARSI	4.49E-30	0.34	3.64E-116	0.38
LHX8	1.01E-54	0.45	4.07E-116	0.38
DLX5	1.03E-26	0.32	1.39E-115	0.38
CORO1C	1.37E-48	0.42	2.61E-115	0.38
IGFL2	3.30E-30	0.34	1.11E-114	0.38
GPC6	3.57E-41	0.39	9.82E-114	0.37
TGFBI	8.56E-50	0.43	8.16E-113	0.37
TNFSF9	1.20E-33	0.35	2.27E-112	0.37
DEPDC7	1.51E-32	0.35	2.54E-111	0.37
JPH3	6.76E-32	0.35	1.23E-110	0.37
HOXA11-AS	3.20E-24	0.30	1.86E-110	0.37
B4GALNT1	5.49E-31	0.34	5.74E-110	0.37
WNT2	1.09E-36	0.37	1.59E-109	0.37
IBSP	1.56E-33	0.35	2.97E-108	0.37
SEMA7A	1.39E-33	0.35	4.86E-108	0.37
ROS1	1.76E-40	0.39	3.99E-107	0.36
KIAA1549L	4.35E-42	0.40	1.58E-106	0.36
LIMS1	2.73E-35	0.36	2.26E-106	0.36
C10orf55	4.89E-38	0.38	7.00E-105	0.36
TLL2	6.92E-38	0.38	1.05E-104	0.36
GBX2	5.80E-35	0.36	3.84E-104	0.36
TNFSF4	9.81E-33	0.35	5.68E-104	0.36
CHRNA1	2.77E-34	0.36	6.15E-104	0.36
SLC11A1	1.28E-24	0.30	1.27E-102	0.36
ADAMTS14	1.42E-25	0.31	6.20E-102	0.36

IGFBPL1	1.12E-29	0.33	1.48E-101	0.35
ITGA5	3.12E-33	0.35	1.61E-100	0.35
TUBB3	1.89E-30	0.34	2.37E-100	0.35
MFAP5	2.48E-27	0.32	9.77E-100	0.35
MURC	7.42E-42	0.39	2.09E-99	0.35
COL12A1	7.03E-27	0.32	1.80E-98	0.35
NXN	2.49E-25	0.31	1.82E-98	0.35
TCHH	1.55E-24	0.30	1.02E-97	0.35
PXDN	3.75E-26	0.31	2.07E-97	0.35
GALNT13	1.23E-33	0.35	2.46E-97	0.35
IGFL3	1.22E-24	0.30	2.71E-97	0.35
LMO7	2.32E-24	0.30	3.21E-97	0.35
LRRC15	1.41E-28	0.33	4.60E-97	0.35
ADCY7	8.77E-33	0.35	1.05E-96	0.35
THBS2	4.86E-28	0.32	4.66E-96	0.35
TMEM169	2.22E-24	0.30	1.60E-95	0.34
PLOD2	8.10E-29	0.33	2.84E-95	0.34
SPP1	7.24E-29	0.33	4.53E-95	0.34
CTHRC1	6.32E-29	0.33	5.07E-95	0.34
KCNJ15	1.87E-35	0.36	6.03E-95	0.34
KANK4	1.04E-25	0.31	3.52E-94	0.34
CD70	1.89E-34	0.36	6.70E-93	0.34
ATP6V0D2	8.45E-47	0.42	3.08E-92	0.34
SLC36A1	4.73E-48	0.42	1.78E-91	0.34
USB1	5.10E-30	0.34	5.57E-91	0.34
MMP9	1.23E-28	0.33	1.15E-89	0.33
SRPX2	5.15E-29	0.33	2.88E-89	0.33
SERPINB7	8.33E-25	0.30	3.07E-89	0.33
COL8A1	1.57E-25	0.31	4.41E-89	0.33
TUBA1C	2.05E-27	0.32	7.20E-89	0.33
ADAMTS2	6.23E-35	0.36	8.27E-89	0.33
TENM4	6.29E-27	0.32	2.48E-88	0.33
ADAMTS12	2.38E-34	0.36	1.52E-87	0.33
CYP27C1	1.13E-46	0.42	4.52E-87	0.33
ACOT9	8.82E-26	0.31	1.13E-86	0.33
KCNJ6	9.64E-39	0.38	1.41E-86	0.33
KIF26B	8.37E-27	0.32	8.25E-85	0.33
FAM26E	2.07E-27	0.32	1.17E-84	0.32
CLIC4	8.37E-25	0.30	3.66E-84	0.32
ALDH1L2	2.72E-41	0.39	4.60E-84	0.32
GPR1	1.63E-24	0.30	1.20E-83	0.32
NID2	2.11E-29	0.33	1.42E-83	0.32
RAI14	1.16E-27	0.32	9.14E-83	0.32
GUCA1A	2.06E-45	0.41	3.09E-82	0.32
AQP9	3.88E-51	0.43	1.17E-81	0.32
MMP8	1.83E-34	0.36	2.35E-81	0.32
PTPRD	2.04E-27	0.32	7.66E-81	0.32
GARS	3.47E-29	0.33	1.11E-80	0.32

SALL1	3.95E-27	0.32	7.00E-80	0.32
SPSB4	6.66E-25	0.31	8.12E-80	0.32
HK3	1.43E-31	0.34	1.04E-79	0.32
CCNE1	1.33E-26	0.32	1.56E-79	0.32
COL5A1	7.67E-30	0.33	1.85E-79	0.32
ARNTL2	4.83E-28	0.32	9.24E-79	0.31
ACTR3	2.72E-35	0.36	2.19E-78	0.31
HEPH	6.94E-25	0.31	1.56E-77	0.31
SLC35D3	2.26E-28	0.33	1.70E-77	0.31
TMEFF1	6.15E-27	0.32	6.28E-77	0.31
GDNF	7.26E-34	0.36	1.11E-76	0.31
DYRK2	5.18E-33	0.35	2.05E-76	0.31
RAP2B	2.23E-37	0.37	3.47E-76	0.31
PLS3	4.09E-26	0.31	5.77E-75	0.31
KDELC1	1.18E-25	0.31	1.22E-74	0.31
CBX2	5.15E-32	0.35	3.67E-74	0.30
COL5A2	7.13E-26	0.31	6.76E-74	0.30
IGLON5	2.37E-31	0.34	3.97E-73	0.30
CTSL	4.86E-26	0.31	1.48E-72	0.30
MCU	9.43E-25	0.30	2.66E-72	0.30
ACBD4	6.66E-31	-0.34	3.08E-73	-0.30
UBXN10	4.29E-29	-0.33	3.01E-74	-0.30
TMEM25	2.07E-29	-0.33	2.57E-74	-0.31
XPA	2.27E-25	-0.31	1.31E-74	-0.31
ZNF540	1.06E-24	-0.30	1.69E-77	-0.31
PIGH	4.49E-28	-0.32	8.09E-78	-0.31
CAMLG	7.30E-29	-0.33	5.92E-78	-0.31
MOAP1	7.19E-30	-0.33	5.35E-78	-0.31
PGPEP1	2.13E-31	-0.34	2.97E-80	-0.32
BCL2	1.24E-24	-0.30	1.73E-80	-0.32
TAPT1	6.72E-25	-0.31	1.93E-82	-0.32
ZNF396	1.27E-25	-0.31	1.28E-83	-0.32
NOVA1	6.99E-27	-0.32	2.21E-84	-0.32
VAMP2	4.98E-31	-0.34	1.39E-84	-0.32
SYBU	1.21E-27	-0.32	1.75E-85	-0.33
RERG	7.64E-27	-0.32	6.93E-86	-0.33
TSHZ1	8.81E-26	-0.31	6.99E-91	-0.34
CIRBP	4.61E-42	-0.40	2.02E-96	-0.35
APH1B	7.17E-30	-0.33	2.30E-97	-0.35
BTF3	1.07E-29	-0.33	1.60E-101	-0.35
CBX7	2.58E-31	-0.34	1.44E-112	-0.37
LETMD1	7.77E-34	-0.36	1.75E-115	-0.38
